# Cobblestone Throat in a Younger Patient Infected with the Omicron Variant of the SARS-CoV-2 Virus

**DOI:** 10.4269/ajtmh.22-0747

**Published:** 2023-06-26

**Authors:** Genki Inui, Katsuyuki Tomita, Akira Yamasaki

**Affiliations:** ^1^ Division of Respiratory Medicine and Rheumatology, Department of Multidisciplinary Internal Medicine, Faculty of Medicine, Tottori University, Tottori, Japan;; ^2^ Department of Respiratory Medicine, National Hospital Organization Yonago Medical Centre, Tottori, Japan

**Figure 1.**
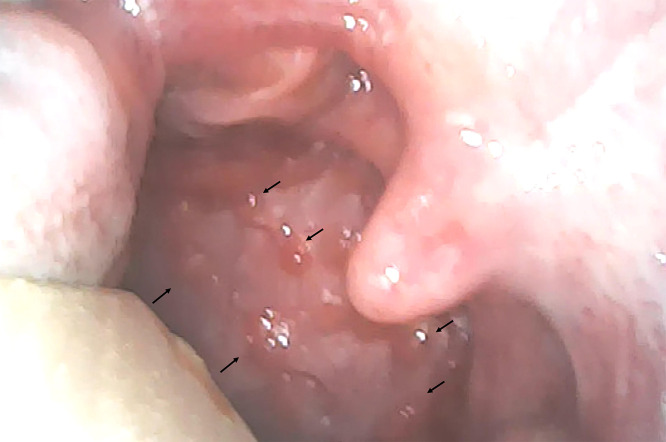
Cobblestone throat at the peritonsillar area on the right side (arrows).

A 21-year-old woman without a history of tobacco use presented to the emergency department (ED) with a 1-day history of an acute scratchy throat and fever. She had an unremarkable past medical history. On physical examination, she had a blood pressure of 116/72 mmHg, a heart rate of 84 beats/minute, a body temperature of 37.6°C, a respiratory rate of 18 breaths/minute, and an oxygen saturation of 98% on room air. No lymphadenopathy was noted. Furthermore, no adventitious sounds were noted on chest auscultation. A Wi-Fi–based flexible endoscope revealed a strawberry-like rough surface, similar to a cobblestone appearance, of the posterior oropharynx (Figure 1). Laboratory examination showed a white blood cell count of 7,400 cells/μL (3,100–8,400), a lymphocyte count of 850 lymphocytes/μL (1,000–4,800), a C-reactive protein level of 1.6 mg/dL (0–0.3 mg/dL), a lactate dehydrogenase level of 349 U/L (120–220 U/L), a ferritin level of 184 ng/mL (20–250 ng/mL), and D-dimer levels of 0.5 μg/mL (0–1.0 μg/mL). Chest X-ray showed no evidence of pneumonia.

She claimed to have been exposed to people with known COVID-19 a few days earlier. The patient tested positive for SARS-CoV-2 via a revere transcription polymerase chain reaction (PCR) nasopharyngeal swab and was diagnosed with COVID-19 infection. This Omicron variant was confirmed by sequences performed as administrative medical examinations. Additional viral testing via PCR-based panel and streptococcal antigen testing was negative. She received a one-time dose of racemic epinephrine aerosol in the ED. Her symptoms improved 1 week later.

**Figure 2.**
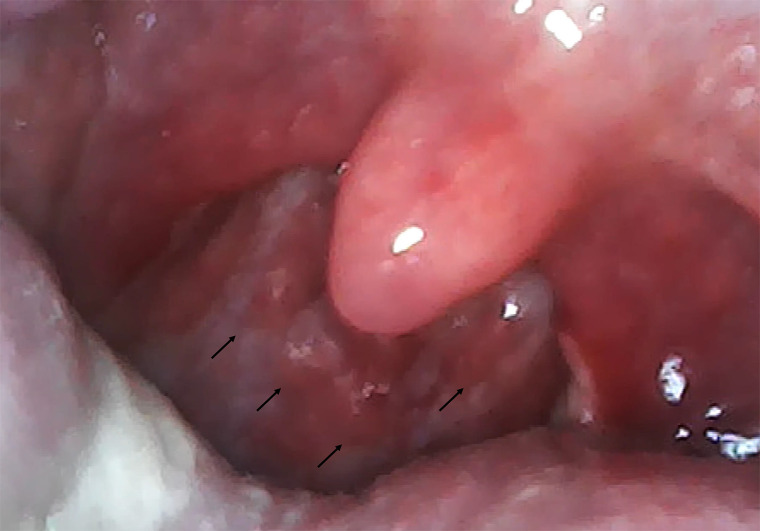
Cobblestone throat of the posterior oropharynx in other COVID-19 cases.

The Omicron strain, first discovered in November 2021, is reported to have a tendency to exhibit strong pharyngeal symptoms in young people.^1^ Although there are reports of severe epiglottitis,^1^ there are few reports of pharyngeal findings. In this case, we found cobblestone throat findings, and later confirmed similar findings in other COVID-19 cases (Figure 2).

The differential diagnosis is influenza from the symptoms and pharyngeal findings. Influenza follicles resulting from influenza infection are well known as pharyngeal findings of viral infection.^2^ The short time from infection to onset is associated with the characteristic follicular formation that is shiny and tense.^2^ In this case, follicles with similar luster were observed, suggesting that the findings may have appeared as a result of the characteristics of the short viral replication time of the Omicron strain.^3^ The finding of cobblestone inflammation of the posterior pharynx in SARS-CoV-2 infection without concurrent influenza infection raises the possibility of a broader differential diagnosis when this physical examination finding is noted, and SARS-CoV-2 and influenza should be differentiated from one another via available diagnostic testing.
